# Characterization of a Listeria monocytogenes plasmid with antibiotic and stress resistance genes

**DOI:** 10.1099/mgen.0.001445

**Published:** 2025-07-07

**Authors:** Clare R. Barker, David R. Greig, Israel Olonade, Craig Swift, Adam Crewdson, Anaïs Painset, Nigel Pittock, Dunstan Rajendram, Gauri Godbole, Paolo Ribeca

**Affiliations:** 1 Gastrointestinal Infections, Food Safety and One Health Division, UK Health Security Agency, London, UK; 2 NIHR Health Protection Research Unit in Genomics & Enabling Data, University of Warwick, Coventry, UK; 3 Gastrointestinal Bacteria Reference Unit, UK Health Security Agency, London, UK; 4 NIHR Health Protection Research Unit in Gastrointestinal Infections, University of Liverpool, Liverpool, UK; 5 Division of Infection and Immunity, The Roslin Institute and Royal (Dick) School of Veterinary Studies, University of Edinburgh, Edinburgh, UK; 6 Food, Water & Environmental Microbiology Service, UK Health Security Agency, London, UK

**Keywords:** antimicrobial resistance, *Listeria*, mobile genetic element, plasmid

## Abstract

Listeriosis is a predominantly foodborne infection causing severe, invasive disease in the immunocompromised, with the causative agent being *Listeria monocytogenes*. While *L. monocytogenes* possesses innate resistance to several classes of antibiotics, acquired antibiotic resistance is low compared with other foodborne pathogens. Conversely, plasmids possessing stress tolerance mechanisms are common and contribute to their ability to persist in food production environments. However, very few have been identified, which also carry antibiotic resistance genes, particularly multidrug resistance regions. We scanned a UK collection of *L. monocytogenes* genomes and detected an isolate with the *dfrD*, *lnuG* and *mphB* genes, encoding predicted resistance to trimethoprim, lincosamides and macrolides, respectively. This isolate also possessed a *Listeria repA* family plasmid replication gene, as well as multiple chromosomal and plasmid-associated stress tolerance factors. Long-read sequencing confirmed the presence of a single 120,420 bp plasmid, which notably is a composite of a highly conserved *Listeria* plasmid backbone with a novel 35 kb resistance region. Comparative genomic analyses revealed that the plasmid-borne resistance region was likely acquired from other Gram-positive species over several events. The island containing the *lnuG* and *mphB* genes has links to porcine and bovine origins, while the *dfrD* gene is located on a Tn*3*-like transposon associated with multiple resistance genes, and both elements can move to the chromosome of *L. monocytogenes* strains from food and human disease. This work furthers the understanding of plasmid-mediated antibiotic resistance as well as the wider circulation of mobile genetic elements among *L. monocytogenes* and other bacterial species within the food chain.

## Data Summary

The complete annotated sequence of isolate 900703 and plasmid p900703_1 described here have been uploaded to GenBank under accessions CP179950 and CP179951, respectively, linked to BioSample SAMN14418420. The raw reads are available in the Short Read Archive under accessions SRR11362024 (short reads) and SRR32162708 (long reads). Table S1 (available in the online Supplementary Material) contains a full list of all publicly available nt sequences that were used for screening, context and comparison.

**Impact Statement**

Plasmids are common in *Listeria monocytogenes*, playing an important role in the species’ capacity to survive in the environment and contaminate a range of foods by providing an auxiliary repertoire of stress tolerance genes. This study provides further evidence of the modular nature of *Listeria* plasmids, more specifically showing that a highly conserved and well-described backbone containing multiple stress tolerance genes can combine with antibiotic resistance-harbouring elements deriving from other genera of bacteria. Widespread genomic surveillance and long-read sequencing where available are powerful tools for investigating the origins of such elements; both have been applied here to reveal important information about the dynamic nature of acquired antibiotic resistance in *L. monocytogenes*.

## Introduction

*Listeria monocytogenes* causes the invasive illness listeriosis among the elderly, immunocompromised individuals and pregnant women and their unborn or newborn babies. The disease is initiated via consumption of contaminated food, leading to intestinal translocation of the bacteria, dissemination and subsequent proliferation within tissues such as the liver, then spread to secondary sites including the central nervous system or placenta. Listeriosis is treatable with antibiotic combinations such as ampicillin and gentamicin, though it requires high doses over a prolonged period due to the intracellular lifestyle of the pathogen, systemic nature of the illness and poor penetration of antibiotics into the cerebrospinal fluid and across the blood-brain barrier [1].

In addition to its potent array of virulence and invasion factors allowing its survival as a facultative intracellular parasite, * L. monocytogenes* is innately resistant to several antibiotics through core genes present on the chromosome of most strains: *fosX* (fosfomycin), *lin* (lincomycin), *sul* (sulfamethoxazole) and *norB* (fluoroquinolones) [2,4]. Alongside this intrinsic resistance, it is not uncommon for *L. monocytogenes* to acquire mobile genetic elements (MGEs) containing antibiotic resistance genes (ARGs) onto its chromosome, such as *tetM* (tetracycline) via a Tn*916*-like transposon [5]. Unlike many other foodborne bacterial pathogens, plasmid-acquired ARGs are a relatively uncommon occurrence among *Listeria* spp.[6], but plasmid presence has previously been implicated in the selection of a resistant strain during antibiotic treatment for diverticulitis, leading to bloodstream dissemination and ultimately endocarditis [7]. Although several ARG-carrying plasmids have been described [7,8], many contain just one resistance gene, with those conferring multidrug resistance (MDR) remaining rarely reported [9,10]. Meanwhile, plasmids containing stress tolerance genes such as biocides and heavy metal resistance genes (BMRGs) are known to be common and widespread among *Listeria* spp. globally, present in around half of all strains of *L. monocytogenes* [11,13]. These plasmids have a modular structure with recombination leading to many permutations of elements encoding tolerance to metals, disinfectants, heat and other stressful environmental conditions. There have been isolated reports of such BMRG plasmid backbones acquiring ARG modules [14].

The rise in whole-genome sequencing (WGS)-based surveillance of pathogens such as *L. monocytogenes* has led to a wealth of genomic data available, much of which remains unexplored, particularly regarding the accessory genome. The UK national reference laboratory for foodborne pathogens receives and performs routine short-read WGS of all clinical isolates of *L. monocytogenes* as well as hundreds of isolates each year from food and environmental samples. We detected an isolate with four ARGs alongside a plasmid replication gene. Here, we characterize the complete sequence of this resistance plasmid and describe its apparent stepwise evolution via the acquisition of elements from other species.

## Methods

Clinical, food and environmental isolates of *L. monocytogenes* referred to the Foodborne Pathogens Reference Service at the UK Health Security Agency are routinely sequenced using Illumina paired-end technology and processed through a bioinformatic workflow that quality checks and trims the reads, assigns a serotype and sequence type and performs typing based on SNPs, as previously described [15]. These reads are uploaded to the Short Read Archive under BioProject PRJNA248549; gene presence in 4,875 genomes (as of 31/12/2022) was confirmed using BLAST+ [16] with reference genes downloaded from GenBank (Table S1).

DNA extraction and long-read sequencing were performed as described previously [17] using the Revolugen Fire Monkey DNA extraction kit (Revolugen, UK) according to the manufacturer’s instructions, the Oxford Nanopore Technologies (ONT, UK) Ligation Sequencing Kit and Native Barcoding kit (SQK-LSK108 and EXP-NBD103) and the GridION FLO-MIN106 R9.4.1 flow cell (ONT). Data was produced in a raw FAST5 format and was base-called and de-multiplexed using Guppy v6.4.6 HAC model (ONT) into FASTQ format. Barcoded samples were re-demultiplexed using Deepbinner v0.2.0 [18]. Run metrics were generated using Nanoplot v1.32.1 [19]. The barcode and y-adapter from each sample’s reads were trimmed, and chimeric reads were split using Porechop v0.2.4 [20]. Trimmed reads were filtered using Filtlong v0.2.0 [21] to generate ~50× coverage of the * L. monocytogenes* genome with the longest and highest quality reads. The 50× coverage filtered FASTQ files were assembled using Flye v2.9.2 [22].

Polishing of the assembly was performed in a three-step process. Firstly, Nanopolish v0.11.1 [23,24] was used with both trimmed FASTQ and FAST5 reads, accounting for methylation and with an input self-alignment created using Minimap2 v2.1 [25] and Samtools v1.7 [26], and secondly Pilon v1.22 [27]. A self-alignment of Illumina reads was used as the query dataset with BWA v0.7.17 [28] and Samtools v1.7 [26]. Finally, Racon v1.2.1 [29] was used, again with a self-alignment of Illumina reads using BWA v0.7.17 [28] and Samtools [26]. The closed, circularized assembly was reoriented to start at the * L. monocytogenes* F2365 *dnaA* gene (NC_002973) using Circlator v1.5.5 [30]; the plasmid was reoriented to *repA* using dnaapler v0.8.1 [31]. Annotation of the resulting plasmid sequence was performed using Bakta v1.9.4 (light database v5.1) for analyses and then PGAP [32] upon upload to GenBank. Visualizations and comparisons with public nt sequences (Table S1) were carried out using BRIG v0.95 [33], Easyfig v3.0.0 [34] and Clinker v0.0.30 [35]. For detailed parameters of all bioinformatic software, see supplementary information.

## Results

### Identification of p900703_1

Scanning our collection of *L. monocytogenes* genomes for ARGs resulted in the detection of an isolate (900703) possessing the *dfrD*, *lnuG*, *mphB* and *tetM* genes, encoding genotypic resistance to the trimethoprim, lincosamide, macrolide and tetracycline classes of antibiotics, respectively, and a plasmid replication gene belonging to *Listeria repA* Group 2 [11,13]. Isolate 900703 belongs to sequence type ST325 and clonal complex CC31 and was sampled in 2020 from a chiller floor within a processed pork food production environment. The genome had no genetic matches within our collection, with the closest isolate being 40 SNPs away. Following long-read sequencing, the resultant assembled genome confirmed the presence of a single 120,420 bp plasmid, which we named p900703_1. Plasmid p900703_1 has an overall G+C content of 36.7 mol% and contains 123 coding sequences, with 2 non-coding RNA genes, a NiCo riboswitch and 2 pseudogenes. Alongside this plasmid, 900703 also possesses within its chromosome the SSI1 stress survival islet [36] and a copy of each of the following transposons: Tn*3*-like (*tnpAR*) Tn*5422* encoding cadmium resistance [37], a Tn*554*-like (*tnpABC*) transposon encoding tolerance to arsenic via an *arsCBADR* operon [38] and the conjugative transposon Tn*916*, which carries the *tetM* tetracycline resistance gene [4].

**Fig. 1.**
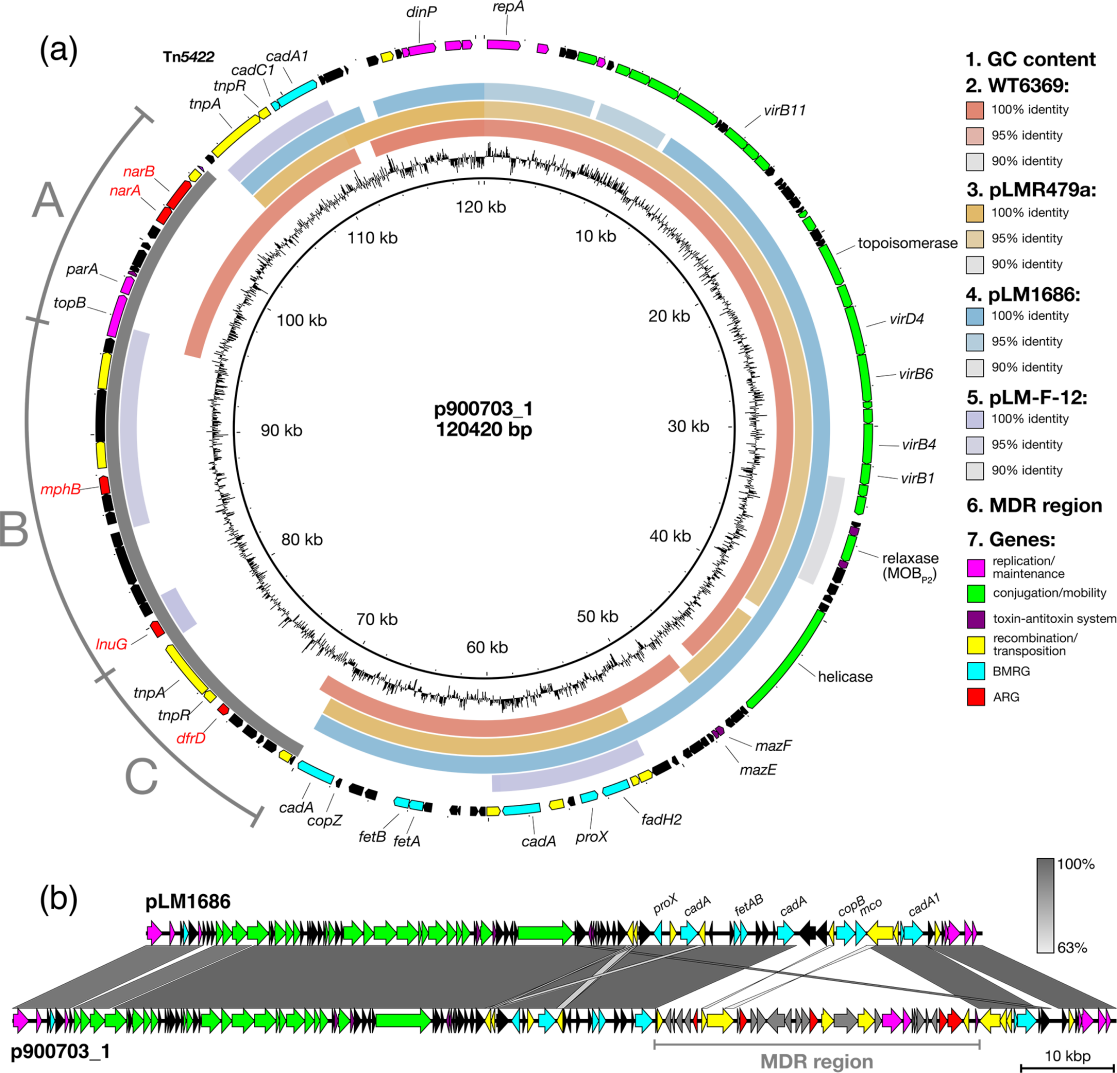
Plots of plasmid p900703_1 showing structure and gene content and relatedness to other plasmids. (**a**) Ring display: GC% (ring 1); blast nt identity to other *L. monocytogenes* plasmids (rings 2–5); location of the MDR region (ring 6). The outermost ring highlights annotated genes of interest and other features of importance, coloured by function. (**b**) blastn sequence similarity plot showing comparison with plasmid pLM1686, including location of the MDR region inserted at the *copB-mco* site, with genes coloured by function as in (a).

To identify genetic matches to p900703_1, we carried out blastn searches against the NCBI non-redundant nt collection. Around 85 kb of the sequence was a close match (>95% nt identity) to *L. monocytogenes* plasmids pLM1686 [39,40] and pLMR479a [41] (Fig. 1). This backbone section of p900703_1 contains the following: the *repA* Group 2 gene; conjugative transfer and mobilization (MobP2 relaxase) genes, which share a layout and 21–51% homology with those of *Bacillus subtilis* plasmid pLS20 [42] and are 99–100% identical to pLM1686 (Fig. S1); *mazEF*-like toxin–antitoxin (TA) system; another Tn*5422 cadAC1* transposon; and several other BMRGs such as the *fetAB* iron export operon and a further two *cadA*-like heavy metal ATPase genes. A noteworthy difference compared with pLM1686/pLMR479a is the absence of the copper tolerance genes *copB-mco* that are typically situated close to Tn*5422* (Fig. 1b). The remaining ~35 kb MDR region with limited similarity to other plasmids contains the three ARGs; initial results from the blastn searches indicated that this section is composed of three sub-regions. No individual chromosomal or plasmid sequences could be identified that possess all three sections together: these were examined separately as Regions A, B and C.

### Characterization of MDR region

**Fig. 2.**
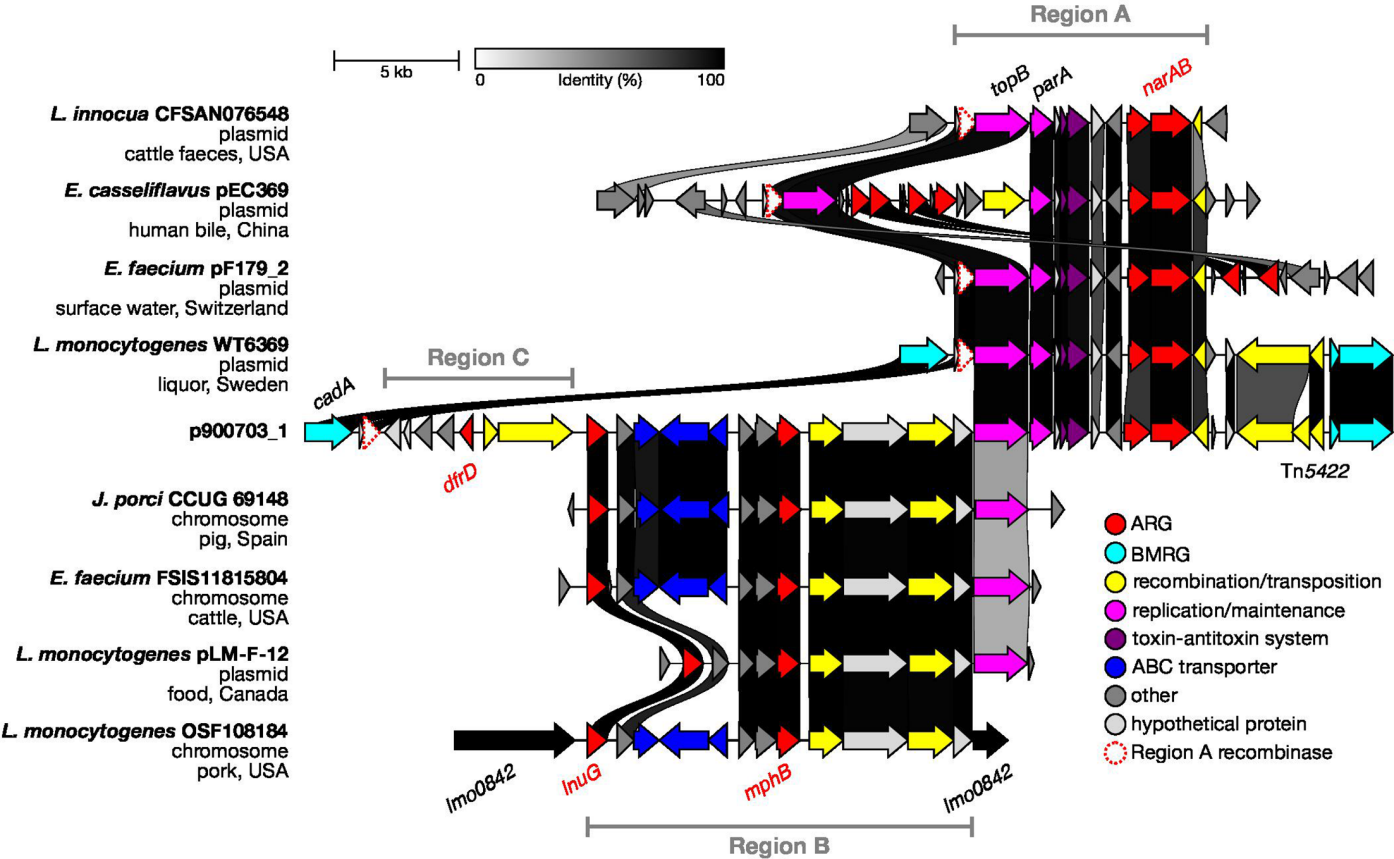
Figure illustrating how p900703_1 Regions B and C have inserted downstream of the recombinase gene (outlined in red) originating from Region A. Sequence similarity of Region A (above) with plasmids from *Enterococcus* spp. and *Listeria* spp. Sequence similarity of Region B (below) with species such as *Enterococcus faecium* and *Jeotgalibaca porci*, with the chromosomal integration in seven *L. monocytogenes* CC20 isolates represented by strain OSF108184.

Expanding the blastn search to include WGS contigs identified *L. monocytogenes* CC8 strain WT6369, which aligned with ~95 kb of p900703_1, additionally including Region A. Region A contains *parA*-like and *topB* genes with a putative TA system, which may contribute to the stability of this region within the plasmid, as well as an ionophore (antimicrobial often used as a growth promoter in animal husbandry) resistance operon *narAB* [43]; none of these genes are present in pLM1686/pLMR479a. Therefore, the plasmid of strain WT6369 appears to be an intermediate form of p900703_1. This region is also similar to one found in an otherwise unrelated *Listeria innocua* plasmid, as well as in *Enterococcus* spp. plasmids where it is situated near other ARGs and displays variability in its layout (Fig. 2). Region A is bracketed by an IS*30* family transposase and a serine recombinase gene, which shares 38% aa similarity with the recombinase located next to the *copB-mco* operon of pLM1686.

The ~16 kb sequence containing the *mphB* and *lnuG* ARGs matched with high overall identity to an element that is present on the chromosomes of *Jeotgalibaca porci* and *Enterococcus faecium*, sampled from pig and cattle, respectively (Fig. 2). Expanding the blastn search to include the WGS contig database detected seven *L. monocytogenes* CC20 isolates from pork that have an identical region incorporated into their chromosomes, where it interrupts *lmo0842* (encoding a cell surface protein). There was also a partial match to the *L. monocytogenes* plasmid pLM-F-12, which is missing the ABC transporter operon but is otherwise identical.

Additionally, we found similar resistance islands located on chromosomes and plasmids of multiple other Gram-positive species. As with those described above, most appear to be isolated from pigs or cattle and related meat products (Fig. S2). Many of the plasmid sequences also have a nearby topoisomerase gene that is similar to *topB* of p900703_1 Region A. Interestingly, one of the porcine *E. faecium* strains possessing *lnuG* also matches several other genes from Region A including the recombinase, further demonstrating the plasticity of this region. Based on the nearby integrase, we propose that Region B of p900703_1 is a mobilizable island that has inserted between *topB* and the serine recombinase gene (both acquired as part of Region A), possibly aided by interspecies homology of the topoisomerase genes.

**Fig. 3.**
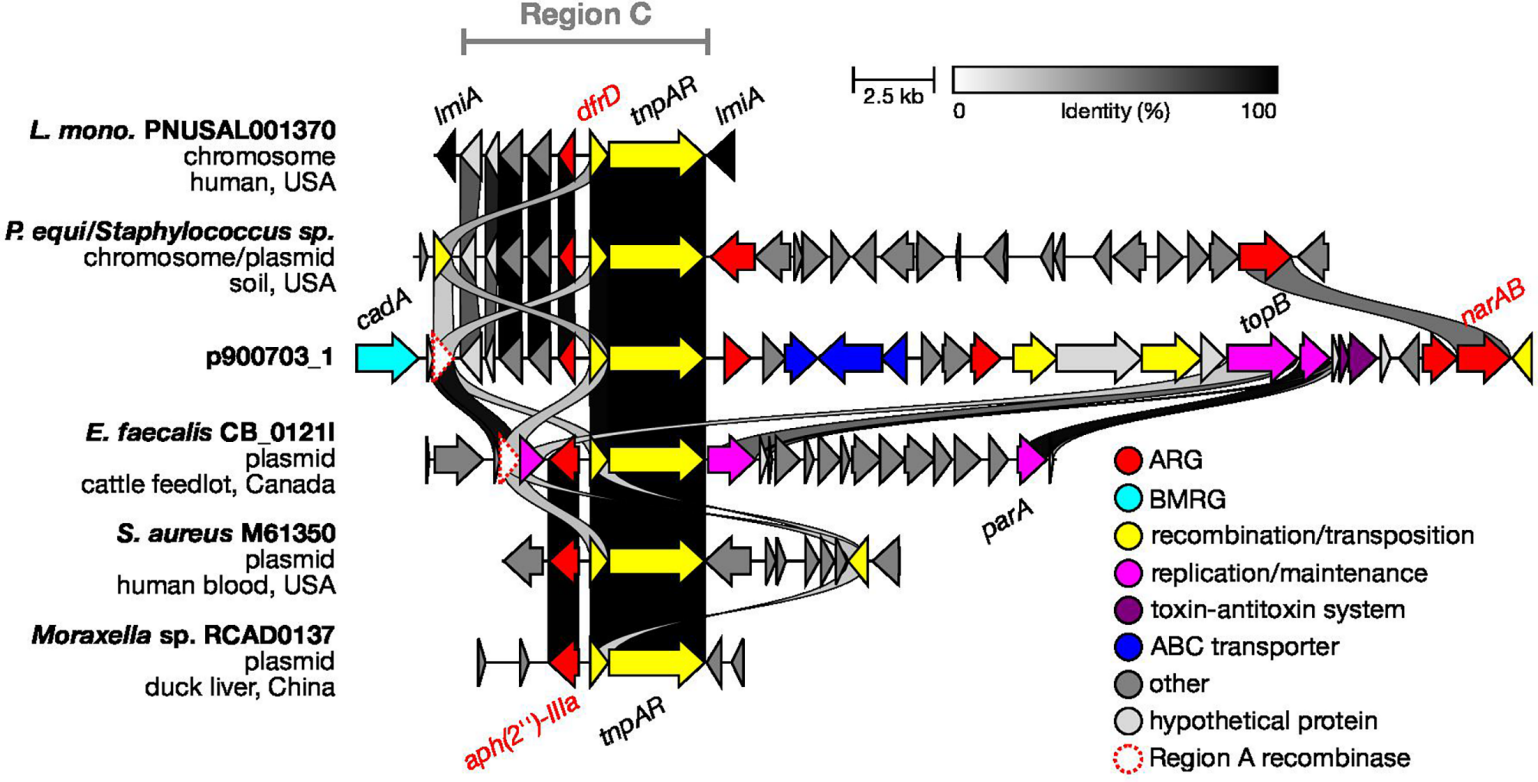
Relatedness of p900703_1 Region C across multiple bacterial species. Above p900703_1: identical elements in *Prescottella equi* and *L. monocytogenes*, with the chromosomal integration in four *L. monocytogenes* CC155 isolates represented by strain PNUSAL001370. Below p900703_1: similar Tn*3*-like elements in more distant species.

The *dfrD* ARG is located on a putative unit transposon between a Tn*3*-like *tnpAR* operon and several other genes that encode an adenylate kinase, a methyltransferase and hypothetical proteins. The *tnpAR* nt sequences display high similarity to transposons from many other genera including *Enterococcus* and *Staphylococcus* (Fig. 3). In these more distant species, the Tn*3*-like transposons are instead associated with the *aph(2″)-IIIa* aminoglycoside resistance gene.

A wider search of WGS contigs revealed that the full ~10 kb Region C is also found in four *L. monocytogenes* CC155 clinical strains isolated from blood in the USA, as well as a *Prescottella equi* contig (however, this assembly is marked as contaminated and, based on blastn searches, may instead be a *Staphylococcus* plasmid) (Fig. 3). In the four isolates of *L. monocytogenes,* the entire transposon has integrated into the chromosome, where it has interrupted the *lmiA* (*lmo1413*) peptidoglycan-binding invasin gene. Interestingly, the *P. equi/Staphylococcus* contig additionally has a nearby ionophore resistance gene. The *Enterococcus faecalis* plasmid also possesses an identical recombinase to p900703_1, as well as *parA* and a disrupted *topB*. These findings provide further evidence of the plasticity of these elements and once again suggest that both the recombinase and topoisomerase are implicated in the acquisition of Region C as well as Region B.

## Discussion

We describe here the sequence of a novel plasmid present in an isolate of *L. monocytogenes* from a pork production environment, comprising a common backbone that has gained multiple resistance genes in addition to its inventory of stress tolerance features. Furthermore, we hypothesize about the sequential formation of this plasmid via the accretion of several regions that can also be found in numerous other bacterial species.

While the majority of antibiotic resistance (e.g. to lincosamides, sulphonamides and fluoroquinolones) in *L. monocytogenes* is intrinsic, there are sporadic reports of *Listeria* spp. horizontally acquiring ARGs via plasmids [44,45] and transposons [46], including a composite transposon containing nine ARGs found in multiple plasmids in China [9,47]. The trimethoprim resistance gene *dfrD* is not uncommon in *Listeria* spp. [44,48, 49], while *mphB* and *lnuG* appear to be rarer occurrences. Other *L. monocytogenes* plasmids possessing the combination of *dfrD-lnuG-mphB* genes have been reported in studies of antibiotic-resistant isolates in France [3] and in a rabbit farm in Portugal [50], both involving ST325 strains. This highlights how the international food and animal trades can lead to the spread of such strains along with their resistance elements. Importantly, the plasmids analysed in both studies were confirmed to confer phenotypic resistance to all three antibiotics.

We observed that the regions surrounding the ARGs were highly similar to elements found in the genomes of multiple other species. Resistance plasmids and transposons found in *Listeria* spp. are often speculated to have originated in other Gram-positive bacteria such as *Enterococcus* and *Streptococcus*, and many have been proven to be transferable to and from these genera [7,9, 10, 44,48]. This is particularly pertinent when considering that genera such as *Enterococcus* regularly occupy the same environmental niches within the food production chain and may even enhance the attachment and biofilm formation capability of *L. monocytogenes* [51]. Additionally, exposure to sub-lethal amounts of disinfectant may co-select for antibiotic resistance and may make *L. monocytogenes* more receptive to acquiring plasmids and other MGEs [52,55]. Such elements could theoretically contain numerous other combinations of ARGs, alongside beneficial features such as more BMRGs (including efflux pumps that further contribute to antibiotic resistance) and even genes providing metabolic effects like wider substrate utilization, thereby raising new issues concerning the control of *L. monocytogenes* within food production facilities.

A noteworthy feature of p900703_1 is the co-occurrence of BMRGs, which are highly prevalent among *Listeria* plasmids [12,13], together with ARGs; this has been rarely reported. In particular, plasmid pLI47-1 [14] has six ARGs together with a similar repertoire of BMRGs to p900703_1, though on a different backbone, while pNH1 and its close relatives pLR1/pLI42/pLI203 contain numerous ARGs and a singular *cadA*-like gene [9,14, 47]. Plasmids such as these provide evidence towards the ability of *L. monocytogenes* to horizontally acquire ARGs without imposing the fitness cost of maintaining a second plasmid or indeed having to lose vital stress response mechanisms. In addition to this, the co-selection of BMRGs alongside ARGs may provide an advantage across multiple environmental niches [56].

The *Listeria* plasmid backbone from which p900703_1 derives is well distributed both geographically and temporally. Plasmid pLMR479a originates from persistent *L. monocytogenes* in Denmark between 1996 and 1999 [41], while pLM1686, which was demonstrated to be self-transmissible and whose conjugation region is almost identical to that of p900703_1, was isolated from multiple strains and serotypes in China between 2001 and 2018 [39,40]. Strain WT6369 – which possesses a putative intermediary plasmid with even higher levels of similarity to p900703_1 – was isolated in Sweden in 2003. The sequence of pLMR479a is also >99% identical to other plasmids including pLmA144 [13,57] and pLmN1546 [58] and appears to be common among * L. monocytogenes*: we detected this same plasmid in ~5% of genomes within our collection (data not shown). Publicly available nt sequences for this family of plasmids reveal that it has been implicated in several outbreaks between 2008 and 2019 that were linked to ready-to-eat meat products [57,59]. Likewise, the MDR plasmid pLI47-1 has a backbone that is very similar to plasmids pPIR00540 and pLM58 [14,60]. This raises interesting questions about the ability of well-established *Listeria* plasmid backbones to acquire resistance modules alongside their already substantial stress tolerance repertoire.

An additional point to consider is the ability of ARG elements that arrive on plasmids to then integrate into the chromosome, whereby they may be maintained and inherited in a vertical fashion. We showed evidence of the *dfrD* element (Region C) being present in the chromosome of clinical strains of CC155 *L. monocytogenes* and *lnuG-mphB* genes (Region B) having been incorporated into the chromosome of CC20 isolates. Interestingly, the *lmo1413* locus at which Region C has inserted is an integration hotspot that is also the site of the LGI3 island [61]. Other studies have correspondingly observed the transposition of MGEs, including large MDR elements, from plasmids into the chromosome of *Listeria* species [14,46, 47]. Continuous positive selection may be necessary to maintain integrated ARG regions in the longer term. However, these regions are likely more stable in the chromosome than on a plasmid, which impose a fitness burden on the host and could undergo segregational loss.

Finally, although it is unlikely that community spread of this particular ARG combination would impact the standard listeriosis treatment of beta-lactams with aminoglycosides, it could potentially have implications in cases where beta-lactam allergies necessitate the use of alternative antibiotics such as co-trimoxazole [5]. Furthermore, horizontal transfer of the plasmid or its MDR region to other bacterial species could have a significant impact on human health due to the loss of multiple classes of antibiotics as treatment options. The combined use of long- and short-read sequencing, as applied in the present study, allows us to monitor these elements as a whole, rather than simply surveying for the presence of ARGs. The multiple lateral genetic links to other species that commonly contaminate the food production chain – as well as the plausible connection between the *lnuG*, *mphB* and *narAB* genes and antimicrobial usage in pigs and cattle – reinforce the strength of considering a One Health approach to reduce acquired antibiotic resistance in *L. monocytogenes* and other foodborne pathogens.

## Supplementary material

10.1099/mgen.0.001445 Uncited Supplementary Material 1.

10.1099/mgen.0.001445 Uncited Table S1.

## Abbreviations

ARG: antibiotic resistance gene
BMRG: biocides and heavy metal resistance gene
MDR: multidrug resistance
MGEs: mobile genetic elements
ONT: Oxford Nanopore Technologies
TA: toxin–antitoxin
WGS: whole-genome sequencing
