# Supplementary material for: Characterization of a Listeria monocytogenes plasmid with antibiotic and stress resistance genes
Source: Microb Genom. 2025 Jul 7;11(7):001445. doi: 10.1099/mgen.0.001445 (PMC12282311; doi:10.1099/mgen.0.001445)
Supplement: Uncited Supplementary Material 1. [file mgen-11-01445-s001.pdf]

Bioinformatics software parameters:

Filtlong: `min_length=1000`, `keep_percent=90` and `target_bases=145Mbp`

Nanopolish: `--methylation-aware=dcm,dam`, `--min-candidate-depth=10` and `--min-candidate-frequency=0.1`

Pilon: `--minmq=0`, `--minqual=0` and `--mindepth=0.05`

Racon: `--error-threshold=0.3` and `--quality-threshold=10`

Supplementary figures:

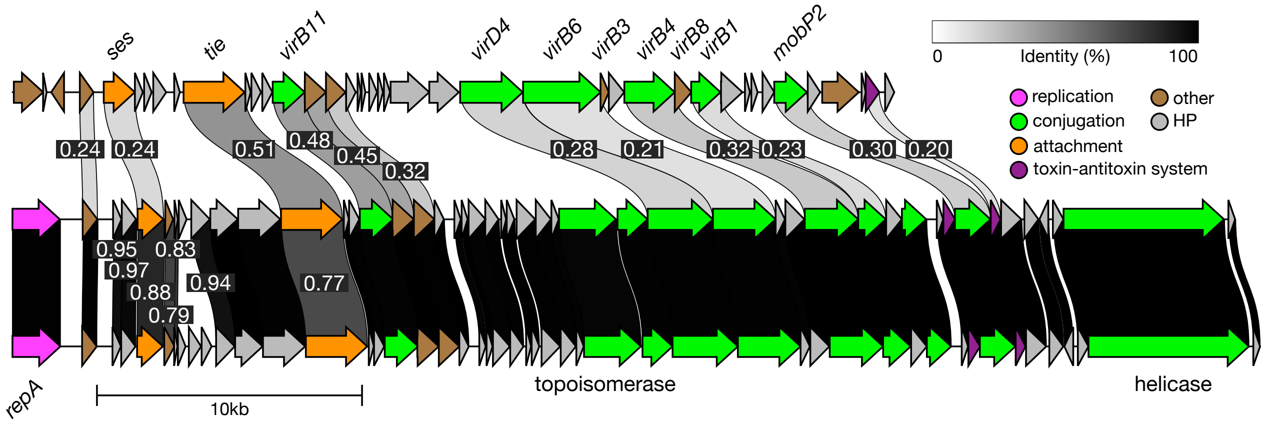

Figure S1) Amino acid sequence similarity between the conjugation regions of *Bacillus subtilis* pLS20[42], p900703_1 and *L. monocytogenes* pLM1686[39,40]. Only identities <99% are labelled. HP, hypothetical protein.

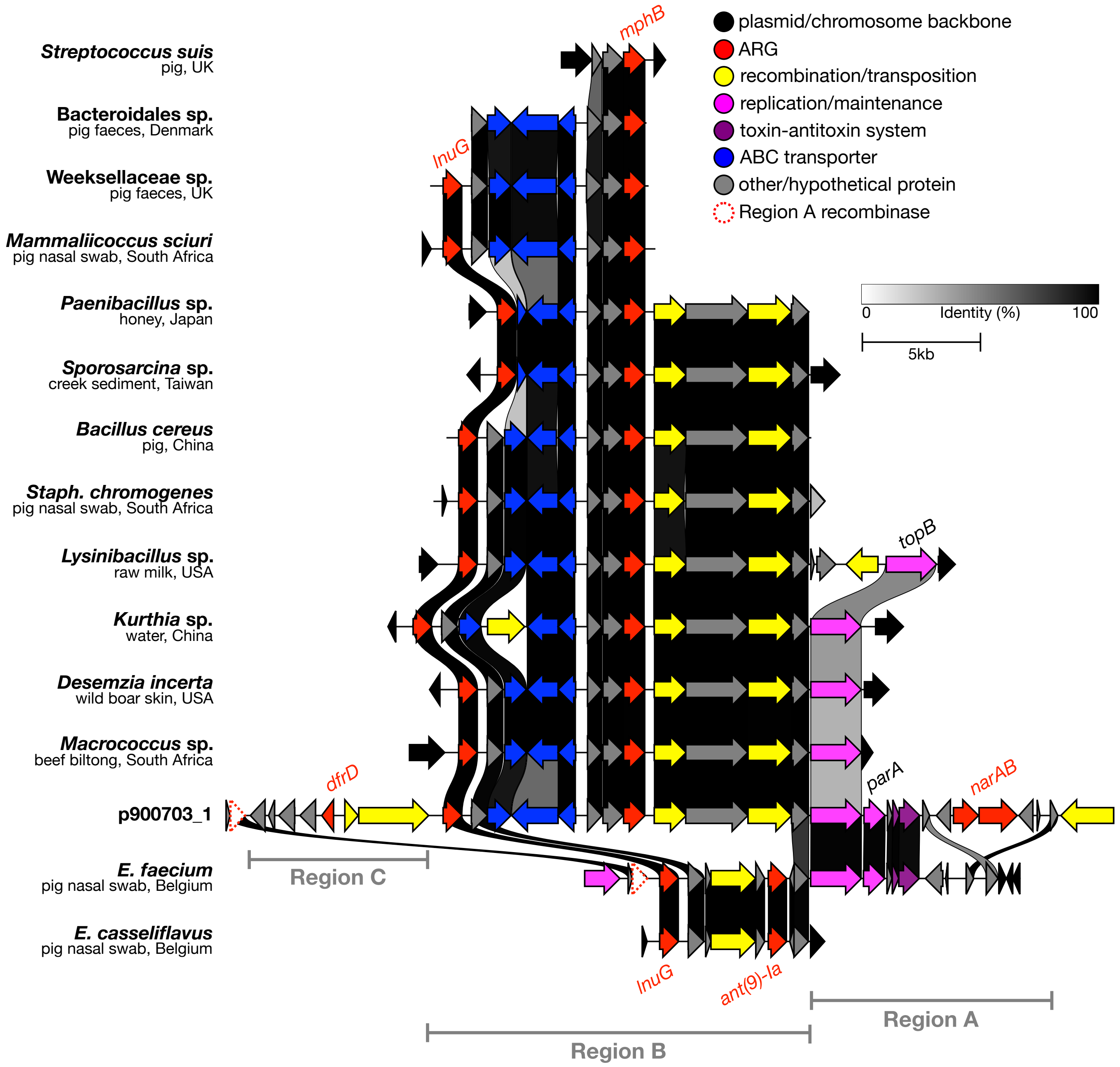

Figure S2) Similarity of Regions A and B with elements in other bacterial species, largely of porcine origin. Above p900703_1: comparison of Region B in other genera of bacteria including similarity with the Region A *topB* gene in some genomes. Below p900703_1: Similarity between *E. faecium* and *E. casseliflavus* strains and p900703_1 Region A and Region B.
